# Evolution of Nanoporous Surface Layers on Gas-Atomized Ti_60_Cu_39_Au_1_ Powders during Dealloying

**DOI:** 10.3390/nano8080581

**Published:** 2018-07-26

**Authors:** Zhenhua Dan, Jiahui Qu, Yulin Yang, Fengxiang Qin, Hui Chang

**Affiliations:** 1 Tech Institute for Advanced Materials, College of Materials Science and Engineering, Nanjing Tech University, Nanjing 210009, China; qujiahuijy@njtech.edu.cn (J.Q.); dan9506@gmail.com (Y.Y.); 2 The Synergetic Innovation Center for Advanced Materials, Nanjing Tech University, Nanjing 210009, China; 3 School of Materials Science and Engineering, Nanjing University of Science and Technology, Nanjing 210094, China; fengxiangqin@njust.edu.cn

**Keywords:** gas-atomized powder, porous material, percolation dissolution, intermetallic phase, dealloying

## Abstract

Nanoporous golf ball-shaped powders with a surface porous layer consisting of fcc Cu and Cu_3_Au phases have been fabricated by selectively dissolving gas-atomized Ti_60_Cu_39_Au_1_ powders in 0.13 M HF solution. The distribution profiles of the Ti_2_Cu and TiCu intermetallic phases and powder size play an important role of the propagation of the selective corrosion frontiers. The final nanoporous structure has a bimodal characteristic with a finer nanoporous structure at the ridges, and rougher structure at the shallow pits. The powders with a size of 18–75 m dealloy faster due to their high crystallinity and larger powder size, and these with a powder size of smaller than 18 m tend to deepen uniformly. The formation of the Cu_3_Au intermetallic phases and the finer nanoporous structure at the ridges proves that minor Au addition inhibits the fast diffusion of Cu adatoms and decreases surface diffusion by more than two orders. The evolution of the surface nanoporous structure with negative tree-like structures is considered to be controlled by a percolation dissolution mechanism.

## 1. Introduction

Nano-scaled pore/ligament bi-continuous nanoporous metals (NPMs) with characteristic length scales from a few nanometers to micrometers, coupled with the extremely high surface area to volume ratio, has resulted in an interest for such applications as actuators [1], sensors [2], catalysts [3], heat exchangers [4], and energy conversion/storage systems [5,6]. Many methods, such as dealloying [7,8,9,10,11,12], ion implantation [13], anodization [14], top-down process in metallic melts [15], template methods [16], etc., have been employed to fabricate nanoporous structures. These NPMs are apt to be fabricated through selective corrosion, so-called chemical dealloying driven by the difference in electrode potentials of constitute elements under free immersion conditions, and electrochemical dealloying (corrosion under applied potential or current) of metallic alloys [7,8,9,10,11,12]. The formation of these NPMs during chemical dealloying undergoes the combined two processes: (1) the selective removal of atoms of one electrochemical-active species from a solid solution to form random porosities at an atomic scale; (2) the rearrangement of noble adatoms at the nanometered scale to result in atomic-scale mixture of interstitials and atoms of the remaining species into ligaments and voids [17,18]. Among NPMs, nanoporous gold (NP Au) is a representative and is fabricated from binary or multi-component alloys, such as Ag–Au [1,7,9,19] and Ag-Au-Pt [20,21,22,23]. These NP Au have revealed a high density of surface defects (atomic steps and kinks) on the curved surfaces of NP Au ligaments, which are confirmed to be active sites for catalysis [24]. Since gold reserves are very rare and the price is relatively high, the cost-effective NP Cu with ultrafine porosities similar with that of NP Au attempts to fabricate and fulfill the functions of NP Au or Au nanoparticles. A number of studies related to the fabrication of NP Cu have been conducted on several alloys, such as amorphous Ti-Cu alloys [25,26], nanocrystalline Ti-Cu alloys [27], Mn-Cu ribbon alloys [28], Al-Cu bulk alloys [29], amorphous Mg-Cu-Y alloy [30], amorphous Ti-Cu-Ni alloys [31], amorphous Ti-Cu-Ag alloys [32], and amorphous Ti-Cu-Au alloys [33]. As has been reported, the three-dimensional NP Cu morphology can be controlled by adjusting the selective corrosion conditions such as the applied potential, dealloying time and electrolyte composition, as well post-dealloying heat treatments which coarsen the structures. On the other hand, the initial microstructures, such as intermetallic phases, crystalline states, and dimensional sizes, play an effective role of the final nanoporous structures of NP Cu [25,26,27,28,29,30,31,32,33]. In nanocrystalline alloys consisting of different heterogeneous phases, i.e., Ti_50_Cu_50_ [27], Ti_59_Al_41_ [34], Zr_2_Ni alloy [35], and Ni-based superalloys [36], the selective dissolution of less noble Ti_2_Cu, α_2_-Ti_3_Al, Zr, and γ-Ni_3_Al is more pronounced than that of the electrochemically stable γ-TiCu, γ-TiAl, Zr_2_Ni, and γ-Ni intermetallics during selective corrosion. The final porous structure inherits the characteristics of the initial microstructure of the precursor alloys [28,34,35,36]. The precursor alloys with optimal chemical compositions and the well-controlled initial microstructures, such as amorphous Ti_60_Cu_39_Pd_1_ and Ti_60_Cu_39_Pt_1_ alloys [37], and Al_7_Cu_4_Ni nanocrystals [38] help with the formation of the ultrafine NP Cu with a characteristic pore size of less than 10 nm, which is similar with reported NP Au. It is worthy to state that the dimensions and shapes of the precursor alloys also affect the final nanoporous structures. Up to date, the different precursor alloys, such as thin film alloy [3,13,39,40,41], nanoparticles [42,43,44,45], nanowires [46,47,48,49,50], ribbons of as-spun alloys [5,8,24,25,26,27,31,32,33,37,38], and bulk alloys [1,9,18,22,38], etc., have been used as the precursors for NPMs. Compared with ion implantation [13], anodization [14], the top-down process in metallic melts [15], and template methods [16], etc., gas atomization is easily handled and free of contamination from the templates, anodizing solutions, or high-temperature melts. Although the dealloying characteristics of the nanoparticles with relatively small particle sizes have been investigated before [42,43,44,45], the gas-atomized precursor powders with a particles size of a few tenths of a micrometer are rarely outlined on the basis of the size effects and the distribution of the intermetallic phases.

The present study focused on the evolution of surface nanoporous layer on gas-atomized Ti_60_Cu_39_Au_1_ powders during selective corrosion from the aspect of the powder size of the precursor powder alloys, distribution of the γ-TiCu or Ti_2_Cu intermetallic phases, propagation of the fronts of the selective corrosion, and the final nanoporous structure of NP Cu and the dominant dealloying mechanisms. 

## 2. Materials and Experimental Procedure

Ti_60_Cu_39_Au_1_, with composition expressed in at%, was gas atomized at CENIM in a confined nozzle atomizer. Gas atomization is a containerless process, where the liquid melt solidifies rapidly at high undercooling, with a cooling rate of 10^3^–10^5^ Ks^−1^. The Ti_60_Cu_39_Au_1_ mother alloy ingots were prepared from Ti (99.99%), Cu (99.999%), and Au (99.99%) by an arc melting furnace. The spraying powders was cooled down in an Ar (99.999%) gas atmosphere of the atomizer. Afterwards, it was collected in air and sieved to achieve separation into two different sizes ranges. This work focused on small, less than 32 μm, and larger 32–75 μm size ranges, hereafter labeled as Powder A and Powder B, respectively. The reference material was amorphous Ti_60_Cu_39_Au_1_ ribbons with 20 μm in thickness and 2 mm in width, fabricated by a melt spinning method. Crystalline states were identified by using an X-ray diffractometor (XRD, Rigaku, Smart Lab, Rigaku Co., Tokyo, Japan) with a CuK_α_ radiation. The dealloying was conducted in 0.13 M HF solution for 10.8 and 54 ks. The microstructure was characterized by a scanning electron microscope (SEM, JEOL 4610, JEOL Ltd., Tokyo, Japan) and a transmission electron microscope (TEM, JEOL, JEM-HC2100, JEOL Ltd., Tokyo, Japan), and the samples for TEM observation were prepared by a focused ion beam (FIB) method. The pore size and the particle size of intermetallic phases were estimated by SEM and TEM observation. The mean sizes of ligaments and nanopores were estimated statistically over 125 particles from SEM and TEM data. The chemical composition of the porous structure was analyzed by energy-dispersive X-ray spectroscope equipped on a SEM (EDX, JEOL 4610, JEOL Ltd., Tokyo, Japan), and energy-dispersive X-ray spectroscope equipped on a TEM (TEM, JEOL, JEM-HC2100, JEOL Ltd., Tokyo, Japan).

## 3. Results and Discussion

### 3.1. Characteristics of the Gas-Atomized Powders

**Figure 1:**
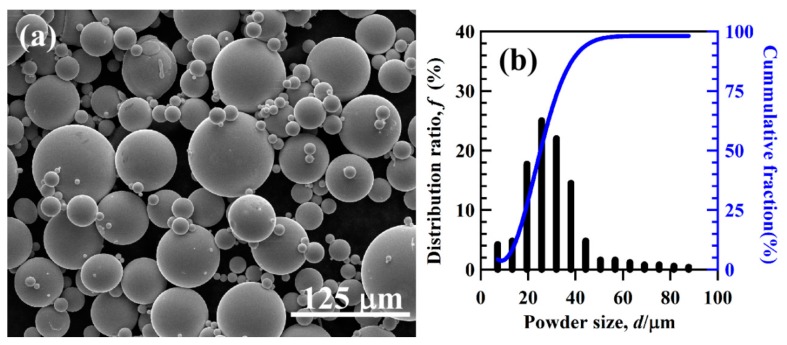
Scanning electron microscopy (SEM) morphology (**a**) and the distribution ratio and cumulative fraction (**b**) of gas-atomized Ti_60_Cu_39_Au_1_ powders.

**Figure 2:**
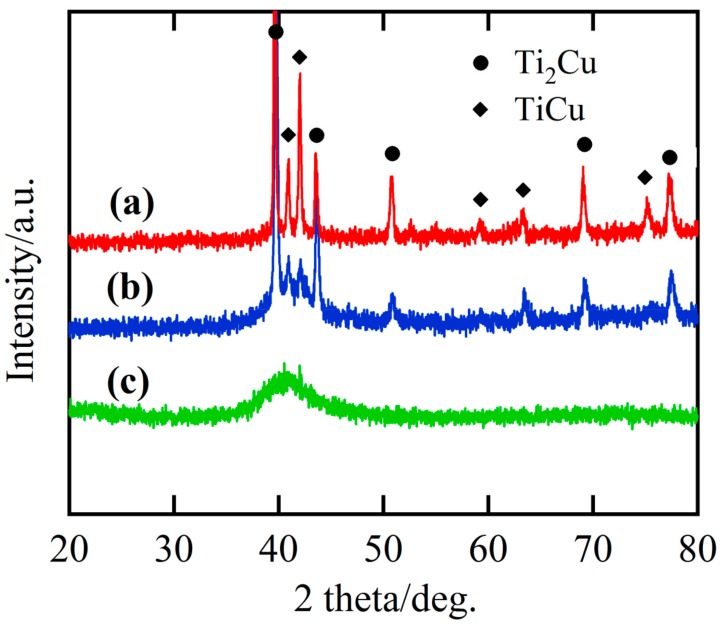
X-ray diffractometor (XRD) patterns of gas-atomized Ti_60_Cu_39_Au_1_ powders with a powder size of 32–75 mm (**a**), under 32 mm (**b**), and as-spun amorphous Ti_60_Cu_39_Au_1_ ribbon (**c**).

The powders prepared by gas atomization process had a spherical shape, as shown in Figure 1a. The size of the gas-atomized powders was from few micrometers to hundredth micrometers. The average powder size of the gas-atomized powders was about 29.4 μm, as shown in Figure 1b. The value of D_10_, D_50_, and D_90_ of the powders were confirmed to be 14.1, 25.1, and 40.3 μm. The powders were screened to group Powder A (<32 μm) and Powder B (32–75 μm). According to the Ti-Cu binary phase diagram [51], intermetallic phases, Ti_2_Cu and TiCu, are possibly precipitated. Some Au-containing intermetallics might precipitate from gas-atomized Ti_60_Cu_39_Au_1_ powders. The XRD patterns of the powders with different powder size in Figure 2a,b had a similar shape and small peaks from Ti_2_Cu (JCPDF Card No: 15–0717), and metastable γ-TiCu (JCPDF Card No.: 07–0114) intermetallic phases were identified after cooling. Several sharp Bragg peaks centered at 2Θ = 40.9°, 42.0°, 63.3°, and 75.1° were assigned to tetragonal TiCu (space group: P4nmm). The rest of the sharp Bragg peaks centered at 2Θ = 39.6°, 43.5°, 50.7°, 69.0°, and 77.2° arose from tetragonal Ti_2_Cu (space group: I4mmm). The XRD pattern in Figure 2c shows that as-spun Ti_60_Cu_39_Au_1_ ribbon had a broad diffraction peak around 2Θ = 41° and another weak diffraction peak around 70–75°. Compared with the three XRD patterns, the high intensity of diffraction peak at 42° and the broad diffraction peak in Figure 2b might be due to the partial amorphous phases in the powders with a size of less than 32 μm. Peaks belonging to the Au-containing phases were not detected. The as-spun Ti_60_Cu_39_Au_1_ ribbons had an amorphous structure and were used as reference materials to evaluate the effect of the distribution of the intermetallic phases on the formation of the nanoporous architecture.

**Figure 3:**
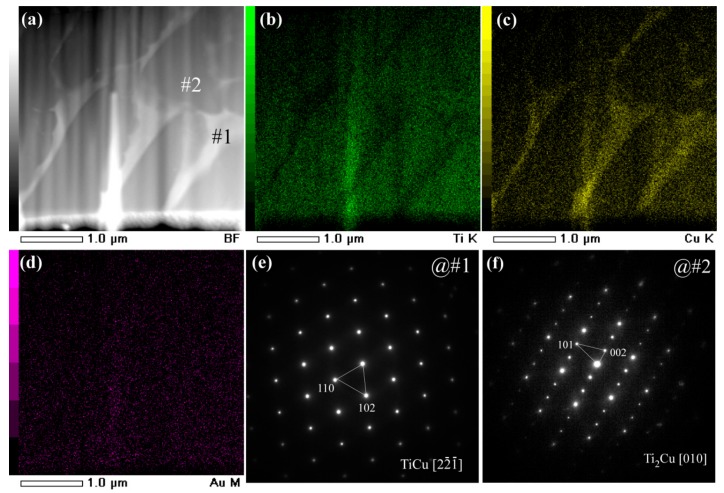
Bright field TEM image (BFI) (**a**) and elemental distribution profiles of Ti (**b**), Cu (**c**), and Au (**d**) of gas-atomized Ti_60_Cu_39_Au_1_ powders with a powder size of under 32 μm. Selected area diffraction pattern (SADP) of corresponding regions at #1 (**e**) and #2 (**f**).

**Figure 4:**
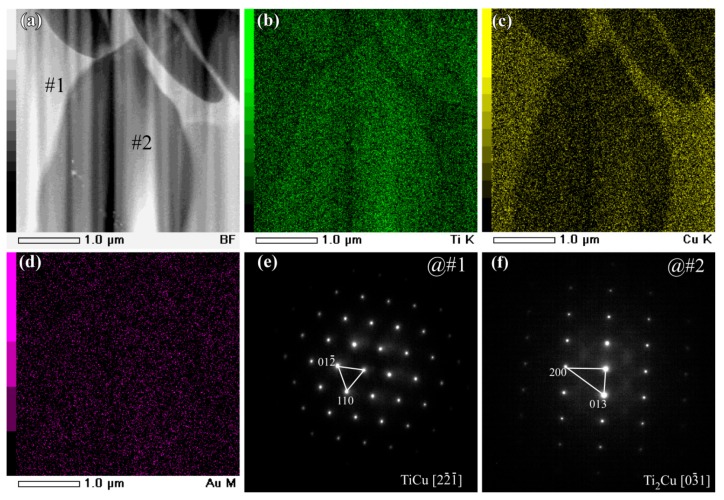
BFI (**a**) and elemental distribution profiles of Ti (**b**), Cu (**c**), and Au (**d**) of gas-atomized Ti_60_Cu_39_Au_1_ powders with a powder size of 32–75 μm. SADP at corresponding regions at #1 (**e**) and #2 (**f**).

The main intermetallic phases in the powders of under 32 mm in Figure 3a were TiCu and Ti_2_Cu intermetallics. The γ-TiCu metastable phase is a phase stable in the high temperature regions. The formation of the metastable TiCu phase was considered to be due to the insufficient cooling rates during the gas atomizing process. On the basis of the elemental distribution mapping data of Ti, Cu, and Au in Figure 3b–d and Figure 4b–d, and selected area diffraction patterns (SADPs) at selected sites, the island-shaped Ti_2_Cu phase (at Site #2 in Figure 3a) precipitated out from the TiCu matrix, and the TiCu phase was distributed continuously (at Site #1 in Figure 3a). The grain size of the Ti_2_Cu in the powders with a powder size of less than 32 μm was comparably smaller than those in the powders with a powder size of 45–75 μm in Figure 4a. The elemental distribution profiles showed that the concentration of Au in the γ-TiCu phase was about 1.3 at%, higher than that in the Ti_2_Cu phase, since the Au elements had a larger solubility in the metastable γ–TiCu phase. According to the phase decomposition of Ti_60_Cu_40_ alloy during the rapid solidification, γ-TiCu phase tended to be distributed continuously and the Ti_2_Cu phase precipitated in the form of the isolated islands [52]. The continuous distribution of TiCu phase and less noble Ti_2_Cu phase was considered to have an effect on the formation of the surface nanoporous layer of the gas-atomized powders. 

### 3.2. Evolution of Surface Nanoporous Layers on Gas-Atomized Ti_60_Cu_39_Au_1_ Powders during Dealloying

**Figure 5:**
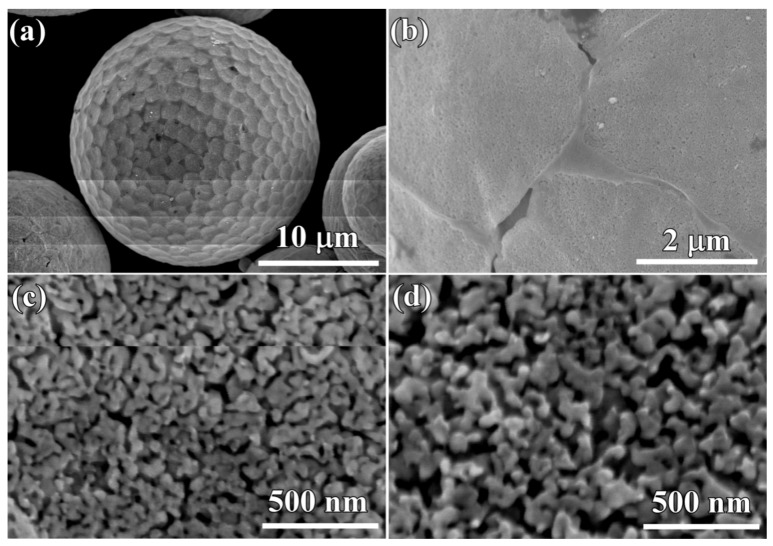
Micrographs (**a**,**b**) and magnified images (**c**,**d**) of Ti_60_Cu_39_Au_1_ powders (Powder A) with a powder size of 20 μm after selective corrosion in 0.13 M HF solution for 10.8 ks.

**Figure 6:**
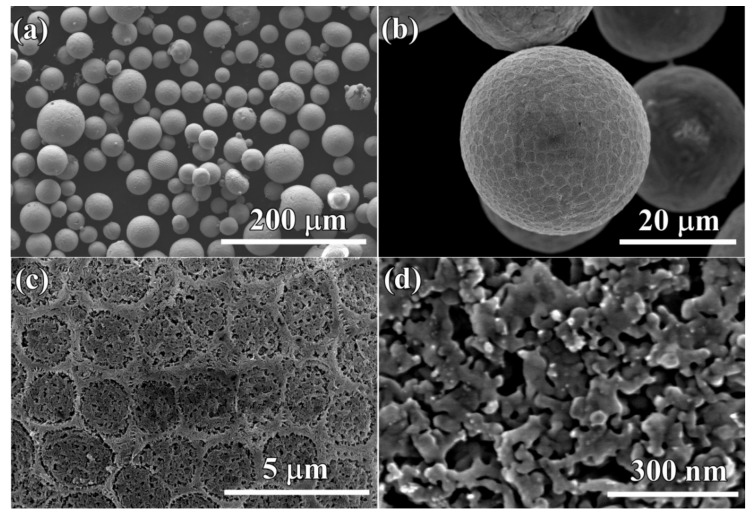
Micrographs (**a**,**b**) and magnified images (**c**,**d**) of Ti_60_Cu_39_Au_1_ powders (Powder B) with a powder size of 35 μm after selective corrosion in 0.13 M HF solution for 10.8 ks.

**Figure 7:**
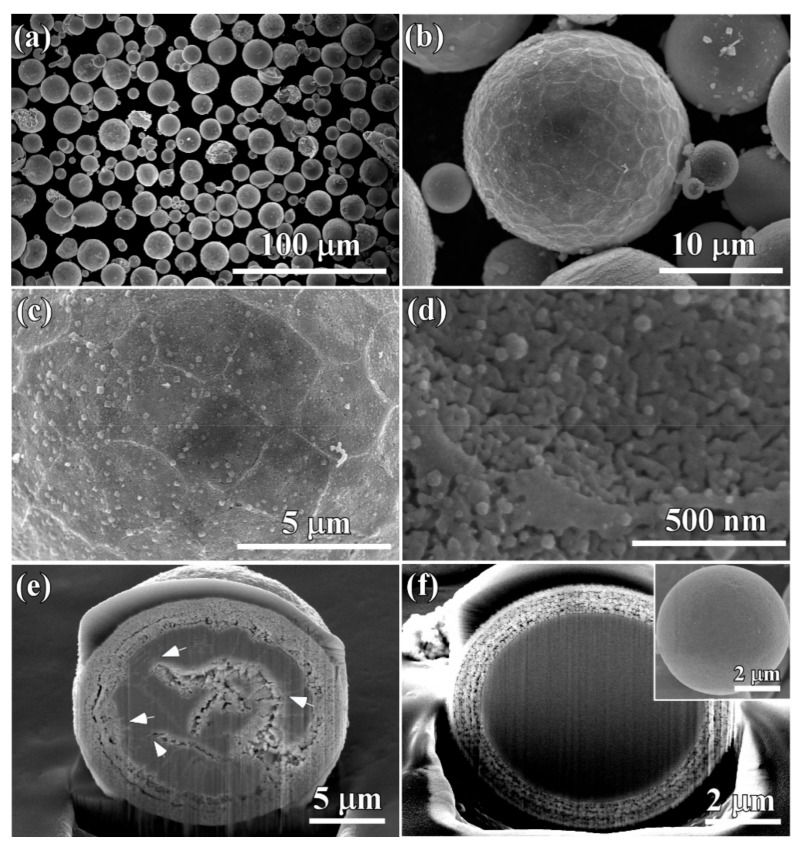
Top-view micrographs (**a**,**b**) and magnified images (**c**,**d**), Cross-sectional micrographs (**e**,**f**) of FIB-edTi_60_Cu_39_Au_1_ powders (Powder A) with a powder size of 18 μm and 7.5 μm after selective corrosion in 0.13 M HF solution for 54 ks.

After the gas-atomized powders with a powder size less than 32 μm (Powder A) were immersed in 0.13 M HF solution for 10.8 ks, surface porous layers formed on the powders. The morphology of single powder with a powder size of 22 μm is shown in Figure 5a. The outline of this powder was similar, with a surface of the golf balls with bucked ridges and heaved pits. In Figure 5b, the porous structure could be seen in both ridge regions and heaved regions. Some voids were formed. The nanopores in the ridge regions had a mean pore size of 15 ± 1.2 nm in Figure 5c, and those in the heaved regions were slightly larger (Figure 5d). Most of the powders with a powder size of 32–75 μm (Powder B) still remained spherical (Figure 6a) after immersion of 10.8 ks in 0.13 M HF solution. However, the magnified SEM images show that they had a similar structure as those in Figure 5a. The distribution of the ridges and pit-shaped regions were macroscopically uniform in Figure 5b, and many pores formed in both ridges and heaved regions. As shown in Figure 5c, the pores with different sizes could be observed on the ridges and pit-shaped regions. The pores in the ridge region where TiCu phases with a width of about 1 μm distributed had a pore size of 16 ± 1.2 nm, and the ligament size was about 26 ± 2 nm. On the other hand, the pores in the pit-shaped regions where Ti_2_Cu phases existed had a larger pore size of 29 ± 2 nm, and the characteristic ligament size was about 35 ± 2.5 nm. After immersion of 10.8 ks, the powders with a larger powder size of 32–75 μm certainly underwent selective dissolution, as shown in Figure 6a. A rough surface formed after dealloying. The single powder with a powder size of about 40 μm in Figure 6b had a similar surface morphology as that in Figure 5a. The detailed information in Figure 6c showed that a bi-continuous structure with ligaments and nanopores formed on the powder surface, although they were bimodal structures. It is worth stating that the pores at the interfacial region between the ridges and heaved regions had a relatively larger pore size. The mean pore size in the heaved region was confirmed to be 18 ± 1.2 nm, and the ligament size was about 32 ± 2 nm as shown in Figure 6d. Many larger voids may have formed due to the collapse of nanoporous structures in the heaved regions. After immersing Powder A for 54 ks, most of the powders still remained as spherical shapes with a rough and porous surface, and many broken powders can be observed in Figure 7a. In detail, the broken powders usually had relatively larger powder size. In the magnified images of Figure 7b, similar morphology with Figure 5b can be seen. The bimodal structure still remained, as shown in Figure 7c. It is worth stating that many particles with different shapes formed on the surface of the dealloyed powders. Most of the particles were cubic, and rest of the particles are polyhedral shaped, such as octahedrons or dodecahedrons. These particles were considered to be Cu because the fast self-diffusion and large diffusion scale of Cu adatoms is apt to accumulate to form this kind of nanoparticles [53]. The characteristic pore size in the heaved regions was confirmed to be 16 ± 1.2 nm, and the ligament size was about 28 ± 2 nm. The cross-sectional images of the powders with a powder size of 18 μm and 7.5 μm are shown in Figure 7e,f. The difference of the internal propagation of the selective dissolution and the penetration behavior of the HF solution was quite obvious. Although the frontier of the selective corrosion was reached in the middle of the powders, some parts were still undissolved after 54 ks of immersion in HF solution. The surface porous layer had a thickness of about 3 μm. There were many arrow-marked intermetallic phases distributed inside the powders. The penetration path of the electrolytes inside the powder seemed to be affected by the distribution profiles of these intermetallic phases. These dendritic precipitates were considered to be TiCu phases on the basis of the TEM data in Figure 3. In most case, the penetration of the electrolyte was prevented by the TiCu intermetallics distributed in the path way of the electrolyte. As shown in Figure 7f, the thickness of the surface porous layer was about 700 nm. Therefore, the penetration rate inside the large powders was much higher than that in the small powders. However, the penetration of the electrolyte for the small powders seemed to be more uniform along the radial direction. There were no large-sized precipitates observed in the matrix. The inset morphology in Figure 7f shows that the outer surface of the small powder with a diameter of 6 μm was uniform without the presence of the ridge-like regions. 

**Figure 8:**
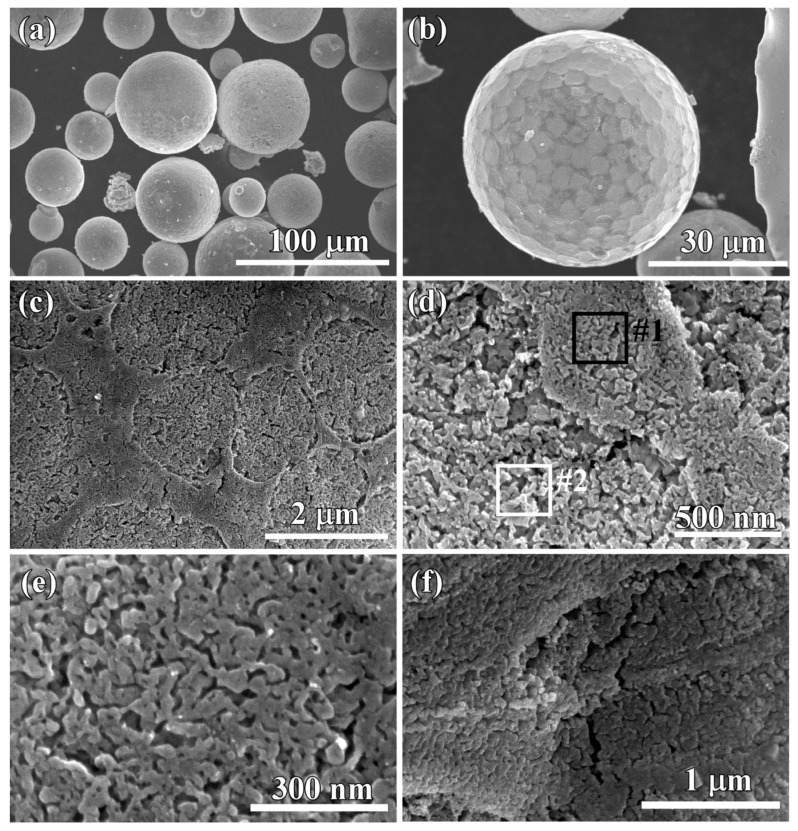
Top-view micrographs (**a**) and magnified images (**b**,**c**,**d**,**e**), Cross-sectional micrographs (**f**) of Ti_60_Cu_39_Au_1_ powders (Powder B) with a powder size of 47 μm after selective corrosion in 0.13 M HF solution for 54 ks.

**Figure 9:**
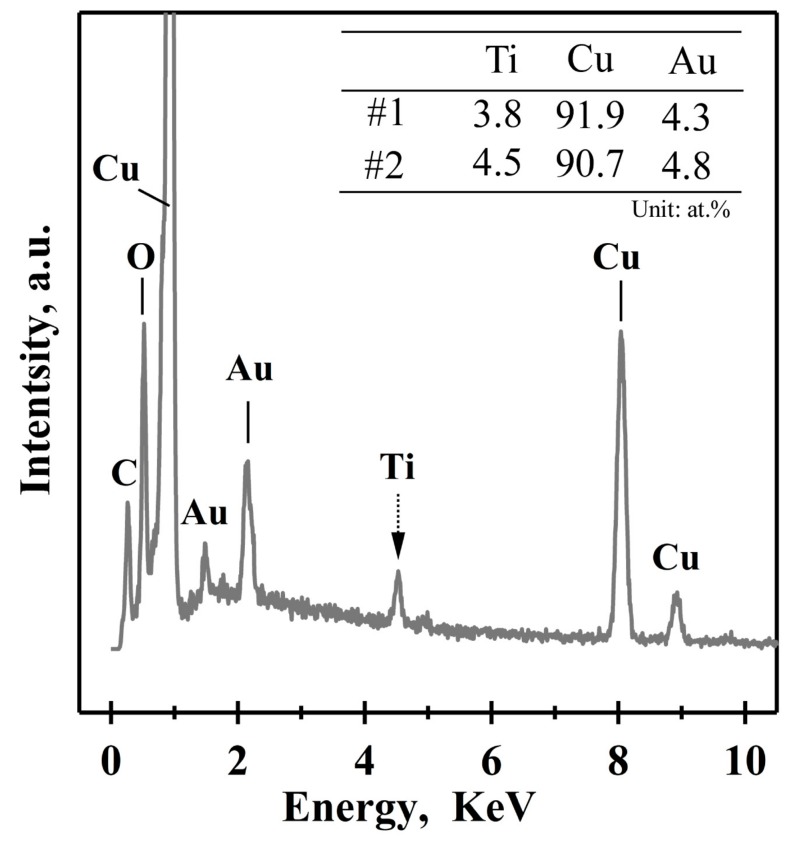
Typical energy-dispersive X-ray (EDX) spectrum of Ti_60_Cu_39_Au_1_ powders (Powder B) with a powder size of 32–75 μm after selective corrosion in 0.13 M HF solution for 54 ks. The chemical composition at Site #1 and #2 in Figure 8c is listed in the inset table.

On the other hand, the Ti_60_Cu_39_Au_1_ ribbon with a thickness of about 20 μm fabricated by melt spinning had an amorphous state, as indicated by the XRD pattern in Figure 2c. Although the cooling rates during gas atomizing were lower than that of the melt spinning, the amorphous powders were able to be obtained when the powder size was small enough. In the present study, the powders with a powder size less than 7.5 μm were considered to be amorphous. The uniform propagation of dealloying for the amorphous alloys was considered to be due to the absence of the intermetallic phases and other defects (i.e., dislocation, stacking faults, and grain boundary). When the powders with a powder size of 32–75 μm (Powder B) were dealloyed after immersion of 54 ks in HF solution, surface porous layers are also formed on the powders, many broken powders can be seen, and the interior structure was also porous, as shown in Figure 8a. The single powder with a diameter of 25 μm was magnified in Figure 8b. The similarity of the surface morphology between those in Figure 5, Figure 6, Figure 7 and Figure 8 was be clearly presented. However, the difference in the width of the ridge region was attributed to the difference in the size of the intermetallic phases in the gas-atomized powders. While the powder sizes became smaller, the size of the γ-TiCu intermetallics at the ridge regions decreased from about 1100 nm for φ47 μm powders in Figure 3c, approximately 920 nm for φ35 μm powders in Figure 8c, down to about 310 nm for φ18 μm powders in Figure 7d. The length of the zig-zag propagation path of selective dissolution in Figure 7e was much larger than 9 μm when the intermetallics were distributed to affect the penetration of the electrolyte. On the contrary, that in Figure 7f was about 700 nm, due to the amorphous powder absence of these intermetallics. All of the facts mentioned above could be considered to be due to the decrease in the powder sizes and the distribution of the intermetallic phases. The nanoporous structure in the heaved regions had a mean pore size of 20 ± 2 nm and a mean ligament size of about 25 ± 2 nm as shown in Figure 8c. The mean pore size and the ligament size in the ridge regions was confirmed to be 16 ± 1.2 nm and 32 ± 2 nm in Figure 8e. A clear height difference was observed between the ridge regions and the heaved regions, and long cracks were apt to form at the interfacial regions in Figure 8f. A typical EDX spectrum is shown in Figure 9. The nanoporous surface layer was mainly composed of Cu, Au, and Ti, and the signals of C and O came from the carbon type and the natural oxidation of the porous powders. The chemical composition of the nanoporous surface layer is listed in the inset table. The Cu concentration was higher than 90 at%, and that of Au was about 4 at%, almost the same at two different sites marked as Site #1 and Site #2. There were about 4 at% residual Ti elements after dealloying in 0.13 M HF solution for 54 ks. The chemical composition of small powders had a similar chemical composition as presented here. The Au concentration of 4 at% in the nanoporous surface layers, four times higher than the gas-atomized powders, indicated that the Cu was partially dissolved during dealloying. 

**Figure 10:**
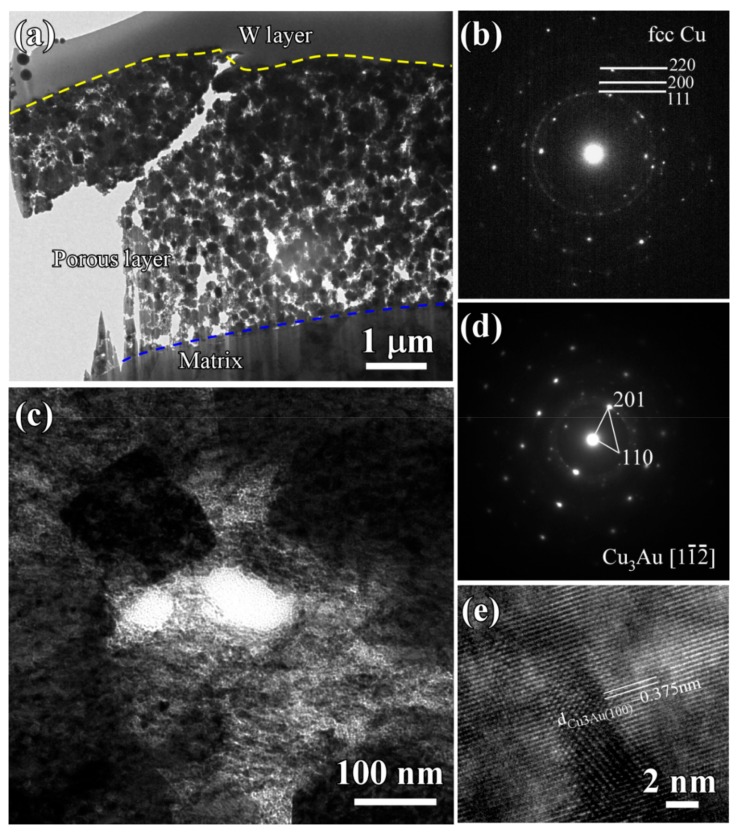
BFI (**a**,**c**), SADP (**b**,**d**) and HRTEM (**e**) of nanoporous layers of Ti_60_Cu_39_Au_1_ powders with a powder size of under 32 μm (Powder A) after selective corrosion in 0.13 M HF solution for 54 ks.

**Figure 11:**
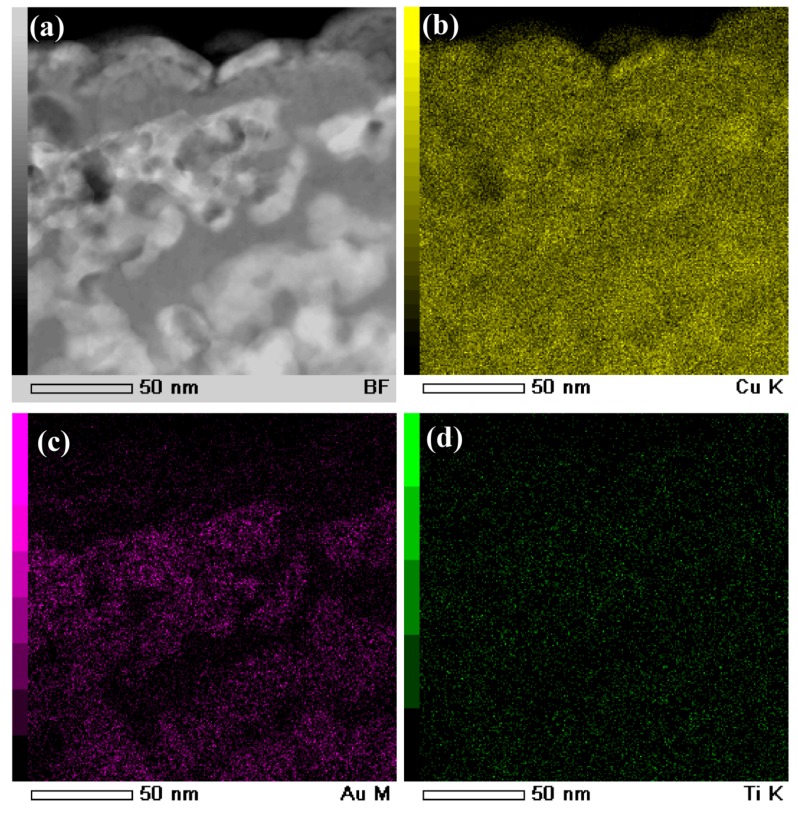
BFI (**a**) and elemental distribution profiles of Cu (**b**), Au (**c**) and Ti (**d**) of Ti_60_Cu_39_Au_1_ powder with a powder size of under 32 μm (Powder A) after selective corrosion in 0.13 M HF solution for 54 ks.

**Figure 12:**
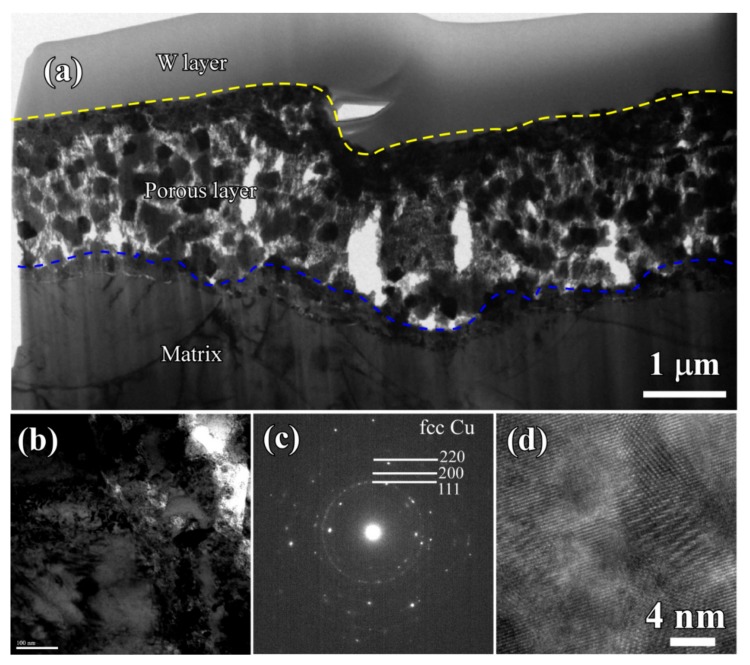
BFI (**a**,**b**), SADP (**c**), and HRTEM (**d**) of nanoporous layers of Ti_60_Cu_39_Au_1_ powders with a powder size of 32–75 μm (Powder B) after selective corrosion in 0.13 M HF solution for 54 ks.

**Figure 13:**
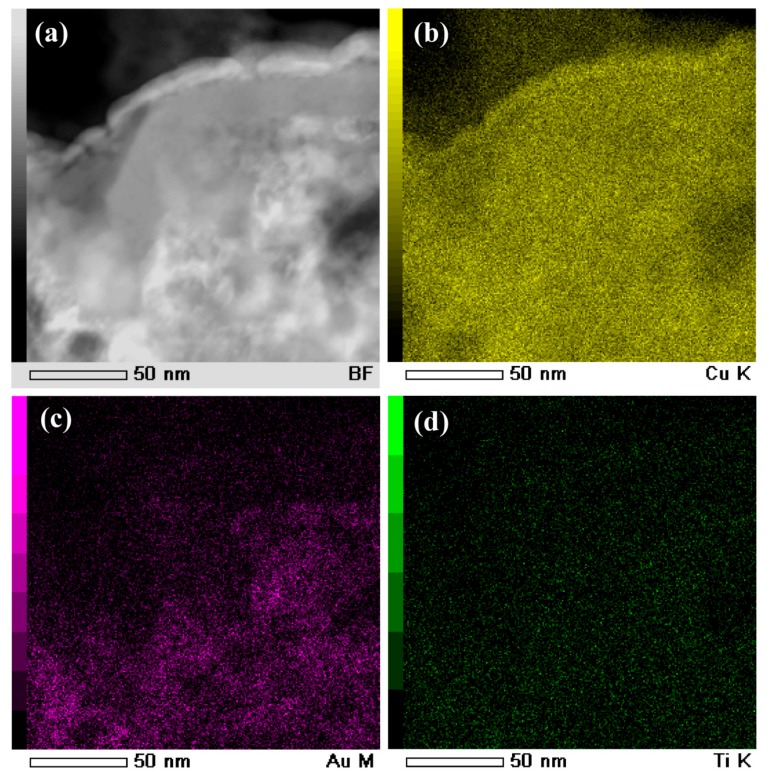
BFI (**a**) and elemental distribution profiles of Cu (**b**), Au (**c**), and Ti (**d**) of Ti_60_Cu_39_Au_1_ powder with a powder size of 32–75 μm (Powder B) after selective corrosion in 0.13 M HF solution for 54 ks.

The cross-sectional bright-field TEM image (BFI) of the surface nanoporous layers formed on Ti_60_Cu_39_Au_1_powders with a powder size of less than 32 μm after dealloying in 0.13 M HF solution for 54 ks is shown in Figure 10a. The nanoporous morphology of dealloyed powders after focused ion beam thinning shows that the distribution of the voids, solid ligaments and secondary phases were distributed almost uniformly in the surface regions. As shown in Figure 10c, the cubic intermetallics, which was confirmed to be fcc Cu_3_Au phase by selected area diffraction pattern (SADP) in Figure 10d, formed after selective corrosion, and coexisted with the main constitute of fcc Cu indicated by the SADP in Figure 10b. Moreover, the high resolution TEM image (HRTEM) in Figure 10e showed that the interplanar distance was 0.375 nm, corresponding to Cu_3_Au (100). The presence of the diffraction rings and the diffraction patterns in Figure 10b and d indicated that the NP Cu ligaments have large-sized and small-sized fcc Cu grains and Cu_3_Au grains, on the basis of the Braggs equations. The average size of the voids was confirmed to be 48 nm, and the distribution ratio of the nanopores with a pore size of 100–200 nm was about 8%. On the other hand, the ratio of the small nanopores with size of 20–100 nm was more than 91%. The profiles of Au elemental distribution in Figure 11c shows that the Au elements were distributed uniformly along the nanoporous ligaments. The NP Cu ligaments also contained mainly Cu and some residual Ti. Meanwhile, the nanoporous morphology of the gas-atomized powders with a large powder size of 54 μm is shown in Figure 12. The SADP in Figure 12c, high-resolution TEM image (HRTEM) in Figure 12d and the elemental distribution profiles in Figure 13 indicated that the surface nanoporous layers also consisted of fcc Cu and cubic Cu_3_Au intermetallic phases. The average size of the voids was confirmed to be 79 ± 5 nm, and the distribution ratio of the nanopores with a pore size of 100–350 nm was about 24%. On the other hand, the ratio of the small nanopores with size of 20–100 nm was more than 76%. The uniformity of the nanoporous structure formed on the powders with smaller powder size was considered to be higher than for the larger powders.

### 3.3. Discussion

The following dealloying mechanisms are well accepted, mainly including (I) “simultaneous” dissolution and redeposition of noble components [54], (II) di-vacancy mediated lattice diffusion of the electrochemically active components to the alloy surface [54,55] and (III) percolation dissolution [56,57,58]. The present Ti_60_Cu_39_Au_1_ powder precursors contained mainly two types of intermetallic phases, γ-TiCu and Ti_2_Cu, and the chemical concentration was suitable for the dealloying thresholds. On the basis of the dealloyed morphologies in Figure 5, Figure 6, Figure 7 and Figure 8, Figure 10 and Figure 11, the percolation dissolution was considered to be the main mechanism during the evolution of surface nanoporous layer on gas-atomized Ti_60_Cu_39_Au_1_ powders during dealloying selective corrosion. For every Ti atoms in Ti_2_Cu intermetallics dissolved from terrace sites on the surface a vacancy is formed at that location. The nanopores on the surface in Figure 5, Figure 6, Figure 7 and Figure 8 formed after selective dissolution of Ti_2_Cu intermetallics, and these nanopores functionalize as the continuous pathways of the electrolyte to invade the inner regions where the Ti_2_Cu intermetallic phases exist. It is noteworthy that the propagating pathways of the selective dissolution in the Ti_60_Cu_39_Au_1_ powders with phase segregation in hundredth nanometer scale are affected by the distribution profiles of intermetallic phases and the powder sizes. As shown in Figure 7e, this diffusion-limited aggregation process underwent and formed dealloyed morphologies consisting of negative tree-like structures or “void-dendrites” penetrating into the solid. With increase of the dealloying time, Cu adatoms and Au adatoms united together to form Cu_3_Au intermetallics and embedded in the surface nanoporous layers (Figure 10 and Figure 12).

On the other hand, the size of the powders also played an important role of the formation of the surface nanoporous layers. As has been reported before, the dealloying behaviour of the Li-Sn and Ag-Au nanoparticles is dependent on the particle size of several tenth nanometers. These particles quickly lose less-noble atoms from the initial surface at fixed potential, and smaller particles tend to lose their surface less-noble elements more quickly because they have a higher fraction of low-coordinated step and kink sites [59,60,61,62]. This is in accordance with the Gibbs-Thomson effect. Kirkendall voiding can occur in principle during dealloying of these particles smaller than 5 nm in diameter, even if absent in the bulk solid owing to the Gibbs-Thompson related depression in the melting point [63,64]. When the particle size is smaller than 300 nm, the surface solid-electrolyte interphase layer forms and hinder the formation of the surface porous structure [59]. In our case, the “void-dendrite” structure in Figure 7e indicates that the mass transport, mainly diffusion, control the propagation of the corrosion front of the selective dissolution. According to the calculation of surface diffusivity in the previous papers [17,33], the surface diffusivity at the ridge γ-TiCu regions was calculated to be 5.14 × 10^−21^ m^2^ s^−1^, and that at the heaved Ti_2_Cu regions was 5.55 × 10^−20^ m^2^ s^−1^. Higher surface diffusivity at the heaved regions causes larger diffusion distance of Cu adatoms to form the relatively rougher nanoporous structure than those at the ridge regions during selective dissolution. However, compared with the nanoporous morphologies formed on amorphous Ti_60_Cu_39_Au_1_ ribbons with a thickness of 20 μm [33], the surface nanoporous structure on Ti_60_Cu_39_Au_1_ powders with a powder size of 32–75 μm became more localized. The powders with a powder size less than 7.5 μm were possible amorphous, as indicated by Figure 2b due to the presence of a broad baseline belonging to amorphous powders. As shown in Figure 7f, the nanoporous surface layer on a powder with a powder size of 7.5 μm looked more uniform in thickness, and the penetration of selective dissolution was clearly different from that of the powders with a larger powder size. Figure 7e depicts the expected dealloyed morphologies at compositions above and below the site percolation threshold, and the penetration rate (significant solid-state mass transport) was much faster than that in Figure 7f. All described above indicated that the percolation dissolution took place for the powders with a large powder size such as Powder B and Powder A with a crystalline microstructure. The percolation dissolution of Ti_2_Cu intermetallic formed the paths (large enough contain enough free volume) for the electrolyte to penetrate the evolving porous structure. As a result, the surface nanoporous layer evolved on Ti_60_Cu_39_Au_1_ powders after percolation dissolution of the Ti_2_Cu intermetallic phases.

## 4. Conclusions

The evolution behavior of surface nanoporous layer on gas-atomized Ti_60_Cu_39_Au_1_ powders with a size of under 32 μm and 32–75 μm has been investigated in 0.13 M HF solution. The gas-atomized powders are crystalline and contain Ti_2_Cu and γ-TiCu intermetallic phases. A surface nanoporous layer consisting of fcc Cu and Cu_3_Au phases have been formed by selectively dissolving Ti_60_Cu_39_Au_1_ powders in 0.13 M HF solution. The propagation of the selective corrosion frontiers occurs in the form of percolation dissolution and is affected by the distribution of the Ti_2_Cu and γ-TiCu intermetallic phases, and the powder sizes. The final nanoporous structure has a bimodal characteristic with a finer nanoporous structure at the ridges and rougher structure at the heaved pits. The highly-crystalline powders with a size of 18–75 μm dealloy faster due to fast mass transport, and these with a powder size of smaller than 18 μm tend to deepen uniformly. The formation of the Cu_3_Au intermetallic phases and the finer nanoporous structure at the ridges proves that the minor Au addition inhibits the fast diffusion of Cu atoms and decrease surface diffusion by more than two orders.

## Supplementary Materials

Supplementary File 1
