# Supplementary material for: Evolution of Nanoporous Surface Layers on Gas-Atomized Ti60Cu39Au1 Powders during Dealloying
Source: Nanomaterials (Basel). 2018 Jul 26;8(8):581. doi: 10.3390/nano8080581 (PMC6116246; doi:10.3390/nano8080581)
Supplement: Supplementary File 1 [file nanomaterials-08-00581-s001.pdf]

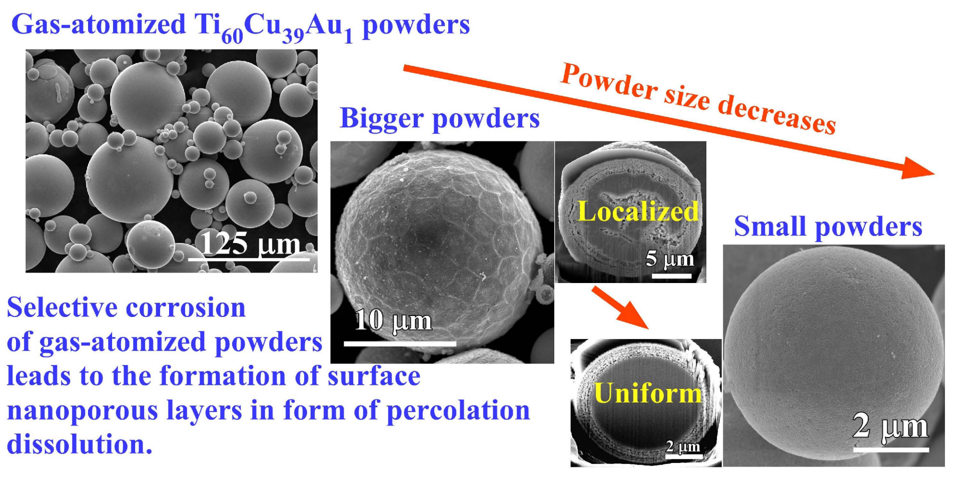

Gas-atomized $Ti_{60}Cu_{39}Au_1$ powders
125 µm
Powder size decreases
Bigger powders
10 µm
Localized
5 µm
Uniform
2 µm
Small powders
2 µm
Selective corrosion
of gas-atomized powders
leads to the formation of surface
nanoporous layers in form of percolation
dissolution.
